# The Role of Vitamin D Deficiency in the Incidence, Progression, and Complications of Type 1 Diabetes Mellitus

**DOI:** 10.1155/2013/148673

**Published:** 2013-03-13

**Authors:** Marlene Chakhtoura, Sami T. Azar

**Affiliations:** Department of Internal Medicine, Division of Endocrinology and Metabolism, American University of Beirut-Medical Center, 3 Dag Hammarskjold Plaza, 8th floor, New York, NY 10017, USA

## Abstract

The “nonclassic” role of 1,25-dihydroxyvitamin D3 (1,25(OH)_2_D_3_) has been recently widely recognized. In type 1 diabetes mellitus (T1D), it plays an immunomodulatory role through the vitamin D receptor (VDR) present on pancreatic and immune cells. Specific VDR allelic variants have been associated with T1D in many countries. Furthermore, vitamin D deficiency has been prevalent in T1D, and the seasonal and latitude variability in the incidence of T1D can be partly explained by the related variability in vitamin D level. In fact, retrospective studies of vitamin D supplementation during pregnancy or infancy showed a lower incidence of T1D. We will review the different mechanisms of the vitamin D protective effect against insulitis and present the available data on the role of vitamin D deficiency in the control, progression, and complications of T1D.

## 1. Introduction

Type 1 diabetes (T1DM) is an autoimmune disease occurring in the pancreatic islets [1]. It accounts for 90% of diabetes in children and adolescents [2]. Its incidence varies considerably worldwide, being highest in Finland and Sardinia [3], probably related to genetic, dietary, and environmental factors that might interfere with its pathogenesis [4]. The annual incidence has been increasing worldwide, possibly related to higher socioeconomic status and degree of urbanization [5]. Recently, there has been appealing evidence on the “nonclassic” role of vitamin D in many autoimmune diseases including rheumatoid arthritis, scleroderma, psoriasis, multiple sclerosis, and also T1DM [6, 7]. In fact, in addition to its skeletal effects and control of calcium hemostasis, 1,25-DihydroxyvitaminD3 (1,25(OH)_2_D_3_) showed potent antiproliferative and immunomodulatory properties [8].

In this paper, we will review the available data on the relationship between vitamin D and T1DM trying to elucidate the immunomodulatory mechanisms of vitamin D on pancreatic insulitis, seasonal and latitude effects, protective effects of supplements on T1DM incidence, complications and progression.

## 2. Immunomodulatory Effect of Vitamin D

1,25(OH)_2_D_3_ plays an immunomodulatory role in the prevention of T1DM, through the vitamin D receptor (VDR) expressed in antigen presenting cells, activated T cells [9], and pancreatic islet *β*-cells [10]; this has been demonstrated in many trials done on nonobese diabetic mice (NOD)—a murine model of human IDDM, spontaneously developing diabetes mellitus (DM)—using 1,25(OH)_2_D_3_ or its analogue (1,25(OH)_2_D_3_, MC1288 (20-epi-1,25(OH)_2_D_3_), or KH1060 (1,25(OH)_2_-20-epi-22-oxa-24,26,27,-trishomovitamin D) [9]. Conversely, 1,25(OH)_2_D_3_-deficient mice were at higher risk of developing DM, with a more aggressive course when deficiency is present early in life [11, 12]. 1,25(OH)_2_D_3_, administered early on, protects against or reduces the severity of pancreatic insulitis via a dual action, on the pancreatic beta cells and on the immune cells [13]. Furthermore, administration of 1,25(OH)_2_D_3_ in combination with cyclosporine A, after the onset of the autoimmune attack, which is known as a prediabetic state, can prevent clinical diabetes [14].

At the level of the pancreatic islets, 1,25(OH)_2_D_3_ decreased in vivo and in vitro proinflammatory chemokine and cytokine expression (e.g., IL6), which are implicated in the pathogenesis of T1DM making *β*-cells less chemoattractive and less prone to inflammation; this results in decreased T cell recruitment and infiltration, increased regulatory cells, and arrest of the autoimmune process [15–17]. Furthermore, 1,25(OH)_2_D_3_ decreases MHC class I expression leading to reduced vulnerability of islet *β*-cells to cytotoxic T lymphocytes [18]. 

**Figure 1:**
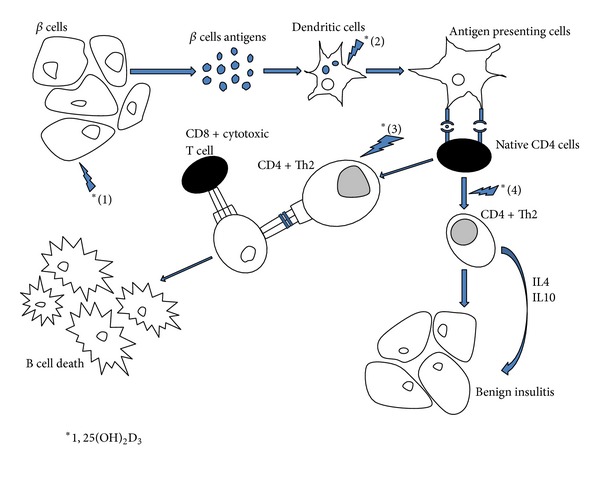


At the level of the immune system, 1,25(OH)_2_D_3_ inhibits the differentiation and maturation of dendritic cells and promotes their apoptosis [19], preventing their transformation into antigen presenting cells which is the first step in the initiation of an immune response [20]. It has been also demonstrated that 1,25(OH)_2_D_3_ restores the suppressor cells, decreases Th1 cytokine production—responsible for *β*-cell death—and shifts the immune response toward Th2 pathway, leading to benign insulitis [21–24]. The addition of 1,25(OH)_2_D_3_ inhibits the production of Il-6, a direct stimulator of Th17 cells [19], implicated in many autoimmune diseases, including T1D [20]. On the other hand, 1,25(OH)_2_D_3_ exerts antiapoptotic effects on the cytokine-induced pancreatic *β*-cells apoptosis. It induces and maintains high levels of A20 gene protein, which leads to decreased nitric oxide (NO) levels. In fact, NO induces directly beta cell dysfunction and death, and, indirectly, through the induction of Fas expression [25]; Fas is a transmembrane cell surface receptor and a member of the tumor necrosis factor (TNF) receptor family. It is stimulated by inflammatory cytokines secreted by islet-infiltrating mononuclear cells. It renders the *β*-cells in T1DM susceptible to Fas-Ligand-induced apoptosis mediated by tissue-infiltrating Fas-Ligand-positive T lymphocytes [26]. Decreasing NO levels leads to down regulation of all the aforementioned mechanisms and allows cytoprotective effects on islet cells. In addition, 1,25(OH)_2_D_3_ has been found to be able to counteract the cytokine-induced Fas expression in human pancreatic islets, both at the mRNA and protein levels, modulating the cascade of death signals and preventing cell apoptosis [27] (Figure 1). 

## 3. Vitamin D Polymorphism 

Vitamin D and its analogues exert their actions through the nuclear VDR which is responsible for transducing the action of the active form of vitamin D, 1,25(OH)_2_D_3_ [28]. The VDR gene is located on chromosome 12q12-q14 in humans [29]. Polymorphisms within the VDR gene may be associated with altered gene expression or gene function [29], and many reports revealed their association with many physiologic and pathologic phenotypes, though inconsistently [30]. Five single nucleotide polymorphisms (SNP) in exon 2 (*Fok*I), intron 8 (*Bsm*I, *Tru9*I, *Apa*I), and exon 9 (*Taq*I) have been defined historically in VDR gene, by the associated restriction enzyme [31]. Association studies of VDR allelic variations and T1D done in many countries, including different populations (southern [32] and northern [33] India, Iran [34], Spain [35], Romania [36, 37], Turkey [38, 39], Hungary [40], Portugal [41], UK, US, Norway [42], Japan [42, 43], Finland [42, 44], Poland [45], Croatia [46, 47], Brazil [48], Uruguay [49], Germany [50–52], Greece [53], Bangladesh [54], Taiwan [55], Chile [56], and Italy [57]) yielded conflicting results; some showed significant association while others failed to reach statistical significance, as shown in Table 1. These different results may be related to differences in ethnic background of the populations studied, interactions with other genetic or environmental factors involved in the pathogenesis of T1DM [34], and possibly differences in ultraviolet radiation exposure [58]. In fact, VDR polymorphisms, with the potential exception of the *Fok*I allele variant which has a differential effect on the immune system [59], may not have any functional effect, so, the *VDR *itself may not be the disease affecting locus but rather a marker locus in linkage disequilibrium with the real disease locus, and the discrepant findings may reflect variable strength of linkage disequilibrium in different populations [41].

The largest meta-analysis to date investigating the association between polymorphisms in VDR gene and T1DM risk found that *Bsm*I polymorphism is associated with a significantly increased risk of T1DM, whereas the *Fok*I, *Apa*I, and *Taq*I polymorphisms do not appear to have a significant association with overall T1DM risk. The *Bsm*I variant B allele (BB or Bb) carriers might have a 30% increased risk of T1DM when compared with the bb homozygote carriers [60].

## 4. Prevalence of Low Vitamin D Level in Type I DM

Given the association between vitamin D and T1DM and the possible role that vitamin D deficiency might play in its pathogenesis, many observational studies have assessed the 25-hydroxyvitamin D (25-OH D) level in T1DM patients (Table 2) and found a significant higher prevalence of 25-OH D deficiency in T1DM patients compared to controls. In Switzerland, in a cross-sectional study, 60–84% of T1DM were 25-OH D deficient [61]. In Qatar, in a case control study, 90.6% of T1DM children versus 85.3% of nondiabetic children had vitamin D deficiency [62]. Similarly, in North India in a case-control study, 58% of T1DM and only 32% of controls had 25-OH D deficiency [63]. In Northeastern US, in a cross-sectional study, it has been found that 15% of T1D patients were 25-OH D deficient and 61% were insufficient, findings inversely associated with age [64]. All these studies showed significantly lower mean 25-OH D level in T1DM compared to controls [62, 63, 65–68]. In addition, in the Diabetes Incidence Study in Sweden (DISS), 25-OH D level was lower in diabetics compared to controls, not only at the onset of diabetes but also at 8-year followup [69].

However, only one study, in Florida, a solar rich region in the United States, found no difference in 25-OH D levels in diabetics (recently or more than 5 months diagnosed) compared to their first degree relatives and controls [70]. 

A pilot study, comparing 25-OH D level in T1DM and type 2 diabetes mellitus (T2DM) showed a higher prevalence of deficiency in T2DM compared to T1DM, and more severe deficiency, independent of age, sex, BMI, and insulin treatment (mean adjusted 25-OH D level 18.1 ± 1.4 ng/mL in T2DM versus 22.9 ± 1.6 ng/mL in T1DM) [71].

## 5. Effect of Latitude on Vitamin D Level and T1DM Incidence

Dermal vitamin D synthesis is a major source of circulating 25-OH D and its metabolites [72]. Sun exposure, strongly related to latitude, predicts 25-OH D level. Many observational studies showed increased T1DM prevalence at northern latitudes where sun exposure is reduced [6].

In Australia, an ecologic analysis of immune-related disorders showed a positive association of T1DM prevalence with both increasing southern latitude of residence and decreasing regional annual ambient ultraviolet radiation (UVR), with an evident threefold increase in prevalence from the northernmost region to the southernmost region [73]. Similar results were found with increasing latitude in Sweden [74] and China [75]. In Norway, a nationwide prospective study showed higher rate of T1DM in southern county and lowest in northern county [76]. 

The EURODIAB collaborative, a large multicenter case-control study including 7 centers, Austria, Bucharest, Bulgaria, Latvia, Lithuania, Luxembourg, North Ireland representing most European countries and Israel, in a report based on 16 362 cases registered during the period 1989–1994 by 44 centers and covering a population of about 28 million children, found a high incidence rate in northern and north western Europe and low in central, southern, and eastern Europe with the exception of Sardinia which presented higher rates than neighboring countries [77], with reverse prevalence, being higher in southern areas [78]. In a worldwide study assessing the pattern of incidence of diabetes in 51 different countries, according to latitude and solar UVR, the incidence rates were higher at higher latitudes and lower ultraviolet B irradiance, adjusted for cloud cover, as inversely associated with incidence rates [79].

Note that interpretation of international correlations is particularly difficult because there are many confounding factors such as affluence and genetic variation. Within country analysis provides probably more precise information [80].

## 6. Seasonal Variability in the Incidence of T1DM

Variability in sun exposure during pregnancy or early developmental stages in infancy has been also suggested as an important environmental factor influencing T1DM onset, possibly related to changes in 25-OH D levels, with highest birth dates of diabetic patients in spring–summer months with an opposite pattern of disease onset peaking in autumn and winter [80]. Consistent results were found in Ukraine (highest variability in western Europe) [81], Sweden [82], Greece [83], Ireland (significant in boys only) [84], Slovenia [85], Germany [86], The Netherlands [87], Britain [88], New Zealand [89], and Sardinia [90]. However, a multicenter cohort study in Europe found no seasonal variations [91]. Similarly, no significant differences in parameters studied in diabetics and controls were detected in Denmark [92]. In a Lebanese T1D population, El Baba et al. showed seasonal variation in glucose control but failed to establish a significant correlation between seasonal changes in 25-OH D levels and HbA1c [93, 94]. In fact, ethnicity may be a confounding factor [95]. Furthermore, none of these studies have shown data about 25-OH D levels, given that they were retrospective. Also, given that viral infections—proven to be involved in the pathogenesis of T1DM—may have also seasonal variations, the evidence of vitamin D involvement in seasonal variations of T1DM needs to be demonstrated with more accurate data.

## 7. Vitamin D Supplementation and Risk of Developing T1DM

Many studies have assessed the effect of vitamin D supplementation during pregnancy, infancy, or early adulthood and the risk of developing T1DM later on in life (Table 3).

The EURODIAB focused on early exposures and risk of T1DM. Vitamin D intake during infancy was assessed by questionnaire or interview (recalled). It showed that vitamin D supplements (given for the prevention of rickets) have a protective effect, even after adjustment for various confounders [96].

Hyppönen et al., in his finish birth cohort study found that vitamin D supplementation of 200 IU daily (as cod liver oil), given to children, was associated with a lower incidence of T1DM during a follow-up period of 31 years [97]. 

In the Norway pilot study, Stene et al. demonstrated a protective effect of vitamin D supplements, only when given as cod liver oil to pregnant women and not when given in other forms of supplementation or when given to children, suggesting a protective effect in utero [98]. However, in his larger case control study, he found a protective effect of cod liver oil when given during the first year of life only and when given ≥5 times per week. No protective effect was detected if vitamin D was given during pregnancy, conflicting results with what have been shown previously by the same group for unknown reasons [99]. 

Similarly, in the DAISY (Diabetes Autoimmunity Study in the Young) study in Colorado, that recruited at birth and followed children at increased risk for T1DM, as determined by HLA-DR genotype or by family history of T1DM, there was a protective effect of vitamin D taken through food only and not as supplements [100]. More recently, Tenconi et al. demonstrated a protective effect of vitamin D given during lactation [101]. 

The ABIS (All Babies in Southeast Sweden) study is a large, prospective, population-based cohort study in Sweden that found vitamin D supplementation, given as drops 10 mcg daily, decreased significantly the incidence of glutamic acid decarboxylase autoantibodies or IA-2A in the offspring at 1 year, but not at 2.5 years [102]. 

Furthermore, the Diabetes Prediction and Prevention study (DIPP), which is a population-based birth cohort of infants at genetic risk of T1DM, showed no significant protective effects of vitamin D whether given with food or as supplements [103]. 

A meta-analysis of the results of observational studies suggests that the risk of T1DM is 29% reduced in those who were supplemented in childhood with vitamin D compared to those who were not [104]. There was some evidence of dose-response effect—higher supplementation resulting in better protection—and the timing of supplementation predicted a favorable response when given between 7 and 12 months, critical period for immunity to become competent [105].

To note that all these studies have several limitations including recall bias, the absence of 25-OH D level, and the absence of quantitative assessment of vitamin D intake; the dose of vitamin D given was not always mentioned. Randomized controlled trials with long periods of followup are needed to establish causality and to suggest the best formulation, dose, duration, and period of supplementation with vitamin D that would allow appropriate protection against T1DM [104]. 

## 8. Possible Explanation of Vitamin D Deficiency in Diabetes

One of the plausible mechanisms of vitamin D deficiency in diabetics is decreased binding proteins; this has been initially demonstrated in diabetic rats [106]. Later on, in humans, it has been found that the urinary loss of vitamin D binding protein (VDBP) is secondary to diminished function or availability of megalin or low-density lipoprotein-related protein 2 (LRP2), correlated with proteinuria. In fact, Megalin is a receptor to many ligands, including albumin, vitamin-binding protein, lipoproteins, hormones, enzymes, and drugs responsible for their reabsorption in the proximal tubule. It facilitates the generation of 1,25(OH)_2_D_3_ following the reabsorption of the VDBP—25OHD complex by via megalin endocytic receptor [107]. Furthermore, a study on pubertal T1DM patients showed altered vitamin D regulatory mechanisms with relative decrease in 1,25(OH)_2_D_3_ plasma concentration and increased 24,25-dihydroxyvitamin D levels in diabetics compared to their healthy counterparts [68]. Note that 25-OH D level upon presentation with diabetic ketoacidosis can be falsely lowered by acidosis and improves with its resolution without any supplementation [108]. 

## 9. Vitamin D Deficiency and Risk of Diabetic Complications

Vitamin D deficiency is associated with increased inflammatory markers in diabetics including CRP, monocyte toll-like receptor (TLR) 2, TLR4, and nuclear factor-*κ*B (NF*κ*B) expression; this might predict increased microvascular complications. However, no statistically significant difference was found in 25-OH D levels in diabetics with microvascular complications compared to those without [109]. On the other hand, another study showed that persistent microalbuminuria is associated with lower 25-OH D levels in T1DM compared to controls [110]. Cardiovascular diseases increased with low 25-OH D levels in the general population [111] but these results have not been specifically studied in diabetics.

25-OH D deficiency has been prevalent upon the initial presentation of T1DM patients who presented with DKA, making it a contributing factor. However, given that levels improved spontaneously after correction of acidosis, the direct contribution of 25-OH D deficiency in the acute presentation of DKA remains controversial [108]. 

## 10. Vitamin D Supplementation Effect on Progression and Control of Diabetes

Given that vitamin D deficiency increases the risk of diabetes development and supplementation showed protective effects, many studies looked at the protective effect of vitamin D on diabetes progression and control. One randomized controlled study aimed to assess the effect calcitriol (given as 0.25 mcg every other day) compared to nicotinamide, within 4 weeks of diabetes diagnosis, on the preservation of *β*-cell function; it showed no improvement in C-peptide and HbA1c levels but significantly lower insulin doses in the calcitriol-treated group [112]. Even when the dose of calcitriol was increased to 0.25 mcg daily and after a followup of 2 years, there was no protective effect of such supplementation on C-peptide levels [113]. Conversely, in LADA patients, when calcitriol (0.5 mcg daily) was added to insulin, it showed stabilization or improvement in fasting and 2 h after 75-g glucose load C-peptide level at 1 year, especially in those whose diabetes duration was less than 1 year [114]. Similarly, in a study in Saudi Arabia, vitamin D_3_ supplementation to T1DM patients who were deficient showed improvement in glucose control (with significantly lower HbA1c) when 25OH D level reached >75 nmol/L at 12 weeks [115].

## 11. Guidelines of Vitamin D Supplementation in Children

The American Academy of Pediatrics and the Canadian Pediatric Association recommended vitamin D supplementation of 400 IU daily, starting the first few days of life [116]. The Institute of Medicine (IOM) recommended that the adequate intake and RDA for children below 1 year of age is 400 IU/d and for all individuals of 1 year to 70 years should be 600 IU/d [117]. It seems prudent to ensure that all infants in the United States and other areas with comparable sunlight exposure receive enough vitamin D, especially in winter [118]. Whether these recommended doses are enough to allow extraskeletal benefits of vitamin D is still unknown.

Until now, no specific recommendations regarding vitamin D supplementation in patients with T1DM or at risk of developing autoimmune diabetes [119] but intakes between 5 mcg daily and the 25 mcg daily, tolerable upper intake level, may be desirable [118].

## 12. Conclusion

1,25(OH)_2_D_3_ immunomodulatory effects have shown significant protection against pancreatic insulitis in animal studies [13–18, 21, 22, 120, 121]. In humans, retrospective analysis and observational studies demonstrated high prevalence of 25-OH D deficiency in patients with T1DM [61–68] and suggested a contributory role in the pathogenesis of T1DM, specially with certain allelic variations of the VDR [32–57]. Conversely, vitamin D supplementation during pregnancy and early childhood decreased the risk of autoimmune diabetes [96–104] and perhaps, even after the onset of diabetes, it may improve glycemic control [114, 115]. Despite all these data, the best dose to be used and the target population in order to decrease the incidence of T1DM have not been yet defined. Abiding by the IOM and the American Academy of Pediatrics recommendations on vitamin D supplementations, at least, improves the 25OH D level.

## Figures and Tables

**Table 1:** VDR gene polymorphism and type 1 DM.

Population	VDR polymorphism associated with IDDM
Bangladesh	*Fok*I, *Bsm*I, *Apa*I, *Taq*I
Brazil	No association
Chile	*Bsm*I, *Apa*I, *Taq*I*¹*
Croatia	*Tru*91, *Fok*I²
Finland	No association
Germany	*Tak*I, *Apa*I, *Bsm*I*¹*, *Tru*I³
Greece	*Fok*I, *Bsm*I, *Apa*I, *Taq*I
Hungary	*Bsm*I, *Apa*I, *Tru*91^4^
India Northern	*Fok*I, *Taq*I
India Southern	*Bsm*I
Italy	No association
Iran	*Taq*I
Japan	*Bsm*I, *Fok*I
Norway	No association
Polish	No association
Portugal	No association
Romania	No association
Spain	*Fok*I
Taiwan	*Bsm*I, *Apa*I
Turkey	*Fok*I
United States	No association
United Kingdom	No association

^1^Combined.

²Dalmatian population.

³In one study, in combination with *Tru*I, VDR polymorphisms were protective against DM type I.

^4^Combined, only in girls.

**Table 2:** Mean 25OH D level in T1DM in different countries.

Country	Mean 25OH D level (nmol/L)
Australia	78.7
Egypt	46.75
Florida	53
Qatar	39.8
Sweden	82.5
Switzerland	45.7
USA (North Eastern)	67

**Table 3:** Vitamin D supplementation and risk of T1DM development.

Author Study design (year)	Country	Population	Vitamin D supplements	Duration	Results (relative risk of T1D with vitamin D supplements)
EURODIAB (no authors listed) Case control study (1999) [96]	7 countries in Europe	746 T1DM and 2188 controls	Vitamin D supplementation during infancy	31 years	0.67 (0.53, 0.86)
Stene et al. Case control study (2000) [98]	Norway	78 T1DM and 980 controls	Cod liver oil to pregnant women	16 years	0.36 (0.14–0.9)
Hyppönen et al. Birth control, prospective study (2001) [97]	Finland	81 T1DM and 10366 controls	Cod liver oil to children during the first year of life (2000IU daily)	31 years	0.22 (0.05–0.89) for regular or irregular vitamin D intake versus no supplements 0.12 (0.03–0.51) for regular vitamin D supplements versus no supplements
Fronczak et al. Cohort study (2003) [100]	Colorado	16 T1DM and 206 controls	Vitamin D supplementation in food, during the third trimester of pregnancy (250IU daily)	4 years	0.37 (0.17–0.78)
Stene and Joner Case control study (2003) [99]	Norway	545 T1DM and 1668 controls	Cod liver oil in the first year of life, at 7–12 months of age (10 mcg daily for at least 5 times per week)	15 years	0.74 (0.56–0.99)
Tenconi et al. Case control study (2007) [101]	North Italy	159 T1DM and 318 controls	Vitamin D supplementation during lactation	29 years	0.33 (0.14–0.81)
Brekke et al. Cohort retrospective and prospective study (2007) [102]	Sweden	8.7% at 1 year and 8.9% at 2.5 years had positive antibodies	Vitamin D supplementation during pregnancy (10 mcg daily)	2.5 years	0.71 (0.17–0.78)
Marjamäki et al. Birth cohort study (2010) [103]	Finland	165 patients with positive antibodies and 4297 control	Vitamin D supplements during pregnancy (mean supplements 5.1 mcg and 1.3 mcg in food, daily)	4 years	No significant protective effect
